# Nivel de conocimientos, actitudes y utilización de medidas preventivas entre los contactos domiciliarios de casos de COVID-19 tras la fase aguda de la pandemia

**DOI:** 10.23938/ASSN.1070

**Published:** 2024-04-10

**Authors:** Vanessa Bullón-Vela, Diana Toledo, Noelia Vera-Punzano, Pere Godoy, Manuel García Cenoz, Jéssica Pardos-Plaza, Jesús Castilla, Angela Domínguez, Iván Martínez-Baz

**Affiliations:** 1 Instituto de Salud Pública de Navarra Pamplona Spain; 2 Instituto de Investigación Sanitaria de Navarra (IdiSNA) Pamplona Spain; 3 CIBER Epidemiología y Salud Pública (CIBERESP) Spain; 4 Universitat de Barcelona Barcelona Spain; 5 Universitat de Lleida Catalonia Spain; 6 Institut de Recerca Biomèdica de Lleida (IRBLleida) Catalonia Spain; 7 Gestió de Serveis Sanitaris Lleida Spain

**Keywords:** Actitud, Conocimiento, COVID-19, Contacto domiciliario, Medidas preventivas, Knowledge, Attitude, COVID-19, Household contact, Preventive measures

## Abstract

**Fundamento:**

Describir el nivel de conocimiento y actitudes sobre la COVID-19 y sus medidas preventivas en contactos domiciliarios de casos de COVID-19 tras la fase aguda de la pandemia.

**Métodos:**

Encuesta a contactos domiciliarios de casos de COVID-19 realizada en centros de salud de Navarra (1) y Cataluña (8) entre mayo/2022 y julio/2023. Se evaluó el uso de medidas preventivas y, mediante 12 ítems, los conocimientos y actitudes frente a COVID-19.

**Resultados:**

Participaron 215 contactos que usaron correctamente las medidas preventivas (>85%), exceptuando mascarilla (35,8%) y distancia interpersonal (47%); >85% mostraron conocimientos adecuados en 5/6 ítems y >80% mostraron actitud positiva en 3/6 ítems. El 54,7% consideró que la COVID-19 influyó negativamente en su vida y el 54,1% que es mejor desarrollar inmunidad enfermando que mediante la vacunación.

**Conclusiones:**

Los contactos domiciliarios mostraron un correcto nivel de conocimiento y una actitud positiva frente a la COVID-19 y sus medidas preventivas.

## Abstract (en)

**Background:**

Assessment of the level of knowledge and attitudes regarding COVID-19 and preventive measures in household contacts of COVID-19 cases after the acute phase of the pandemic.

**Methods:**

Survey among household contacts of COVID-19 cases conducted in healthcare centers in Navarre (1) and Catalonia (8) between May 2022 and July 2023. The frequency of use of preventive measures and the knowledge and attitudes towards COVID-19 were assessed through 12 items.

**Results:**

The study included 215 contacts who correctly used the preventive measures (>85%), except for facemasks (35.8%) and interpersonal distance (47%); >85% showed adequate knowledge (5/6 items) and >80% had a positive attitude (3/6 items). Moreover, 54.7% considered that COVID-19 negatively affected their life and 54.1% that it is better to develop immunity by getting infected than by vaccination.

**Conclusions:**

Household contacts show a correct level of knowledge and positive attitude towards the disease and its preventive measures.

## INTRODUCCIÓN

La COVID-19 es una enfermedad infecciosa causada por el coronavirus SARS-CoV-2 que debido a su rápida propagación fue declarada pandemia por la Organización Mundial de la Salud (OMS) en el año 2020^1^.

Durante la fase inicial de la pandemia se implementaron diversas estrategias de salud pública para el control y prevención de la transmisión del SARS-CoV-2, como el uso de mascarilla y de soluciones hidroalcohólicas, el lavado frecuente de manos y el distanciamiento social, que se complementaron durante el segundo año con la puesta en marcha de un programa de vacunación^1^^-^^4^. En España, estas medidas de prevención se fueron implementando y adaptando de acuerdo a la situación de la pandemia^4^.

Diversos estudios han indicado que los domicilios constituyen un entorno de alto riesgo para la transmisión de la infección y que los contactos domiciliarios presentan un mayor riesgo de contagio debido a su reiterada exposición^5^^,^^6^.

Durante un estado de emergencia sanitaria como la pandemia de COVID-19, los conocimientos, actitudes y la correcta utilización de las medidas preventivas constituyen factores cruciales para controlar la transmisión^7^. Por el momento, no hay evidencia científica sobre el nivel de estos conocimientos tras la fase aguda de la pandemia, momento en el que se produjo una relajación de las medidas preventivas.

En este contexto, el objetivo de este estudio es describir el nivel de conocimiento, actitudes y uso de medidas preventivas entre los contactos domiciliarios de casos de COVID-19 tras la fase aguda de la pandemia.

## MATERIAL Y MÉTODOS

Se diseñó un estudio epidemiológico prospectivo mediante una encuesta telefónica a los contactos domiciliarios de casos confirmados de COVID-19. Los contactos se reclutaron entre mayo de 2022 y julio de 2023 a través de nueve centros de salud de atención primaria, uno en Navarra y ocho en Cataluña, representativos de cada comunidad autónoma.

La metodología y cuestionario utilizado en el estudio han sido descritos anteriormente^8^. En resumen, todos los contactos domiciliarios fueron testados mediante una prueba rápida de antígenos para SARS-CoV-2 tras la notificación del caso confirmado, y mediante reacción en cadena de la polimerasa (PCR) siete días después en aquellos contactos que dieron negativo en la primera prueba.

El cuestionario utilizado^8^ fue diseñado a partir del utilizado por Hong Xu y col^9^ y Pascal Geldsetzer^10^, y basado en las recomendaciones realizadas por la OMS, el Centro Europeo para la Prevención y Control de Enfermedades (ECDC) y el Ministerio de Sanidad español^1^^-^^4^. Su estructura se evaluó mediante una prueba piloto^8^.

El cuestionario constaba de siete secciones. Las cuatro primeras recogían información específica sobre:

- Características sociodemográficas: sexo (hombre, mujer), grupo de edad: <18, 18-44, 45-64, ≥65 años;
- Información clínica: presencia de enfermedades crónicas (enfermedad pulmonar obstructiva crónica, cardiopatía coronaria, accidente cerebrovascular, diabetes, enfermedad renal y hepática crónica, entre otras), presencia de factores de riesgo (hábito tabáquico, obesidad e hipertensión arterial);
- Información epidemiológica: uso de mascarilla, frecuencia de lavado diario de manos (ninguna, 1-2, 3-4, >4 veces), existencia de distancia interpersonal y de ventilación;
- Estado de vacunación frente a la COVID-19 (no vacunado, 1 dosis, 2, 3, 4 dosis).

En las tres últimas se preguntaba a los contactos mayores de 18 años sobre los conocimientos y actitudes frente a la COVID-19 y sus medidas preventivas. Cada sección estaba compuesta por seis preguntas que recogían la respuesta mediante una escala Likert de 5 puntos (5: totalmente de acuerdo, 4: de acuerdo, 3: ni de acuerdo ni en desacuerdo, 2: en desacuerdo, 1: totalmente en desacuerdo). Además, las respuestas relativas a los conocimientos y actitudes se agruparon en dos categorías: correctas (4 y 5) e incorrectas (1 a 3), excepto en la pregunta de conocimientos *Desarrollo de caso grave de COVID-19* y en la pregunta de actitudes *Desarrollar inmunidad enfermando de la COVID-19 vs vacunándose*, en las que se consideraron correctas las respuestas 1 y 2. En la pregunta sobre *Susceptibilidad a desarrollar un caso grave* se consideró una actitud correcta si la respuesta era 4 o 5 solamente si el participante presentaba alguna comorbilidad y/o factor de riesgo.

El estudio fue aprobado por la Comisión de Bioética de la Universidad de Barcelona (IRB00003099 - 02/03/2022). Todos los participantes proporcionaron su consentimiento de manera verbal para participar en el estudio. Se excluyeron los contactos domiciliarios con alguna discapacidad cognitiva o auditiva que pudiera impedir completar las entrevistas.

Se realizó un análisis descriptivo de todas las variables en estudio mediante frecuencias absolutas y porcentajes. Se calculó la tasa de ataque secundaria (TAS) para cada una de ellas, a fin de determinar la proporción de casos de COVID-19 ocurridos entre los contactos domiciliarios. Se calculó la proporción de contactos que respondieron correctamente a los ítems relativos a las secciones sobre conocimientos y actitudes frente a la COVID-19 y sus medidas preventivas. Se utilizó la prueba de Chi-cuadrado para la comparación de TAS entre categorías de variables. Se empleó el paquete estadístico SPSS 25.0; y los valores de p <0,05 fueron considerados estadísticamente significativos.

## RESULTADOS

### Características de los contactos y tasa de ataque secundaria

Durante el periodo de estudio se realizó la encuesta a un total de 215 contactos domiciliarios de casos de COVID-19, el 74% en Cataluña. El 51,2% fueron hombres y la mayoría tenían más de 45 años (59,1%) mientras que solo el 20% tenían menos de 18 años; el 27% tenía alguna enfermedad crónica y el 47,4% presentaba algún factor de riesgo (Tabla 1).

**Tabla 1:** Características y tasa de ataque secundaria de los contactos domiciliarios de casos de COVID-19

	Contactos domiciliarios	Infecciones	TAS	p
	n (%)	n	%	
*Grupos de edad (en años)*				*0,001*
<18	43 (20,0)	10	23,3	
18-44	45 (20,9)	18	40,0	
45-64	77 (35,8)	30	39,0	
≥65	50 (23,3)	35	70,0	
*Sexo*				*0,324*
Hombre	110 (51,2)	44	40,0	
Mujer	105 (48,8)	49	46,7	
*Enfermedades crónicas*				*0,001*
No	157 (73,0)	57	36,3	
Sí	58 (27,0)	36	62,1	
*Factores de riesgo*				*<0,001*
No	113 (52,6)	36	31,9	
Sí	102 (47,4)	57	55,9	
*Uso de mascarilla*				*0,627*
No	138 (64,2)	58	42,0	
Sí	77 (35,8)	35	45,5	
*Frecuencia de lavado de manos*				*0,427*
0 veces/día	2 (0,9)	1	50,0	
1-2 veces/día	26 (12,1)	11	42,3	
3-4 veces/día	75 (34,9)	27	36,0	
>4 veces/día	112 (52,1)	54	48,2	
*Distancia interpersonal*				*0,111*
No	113 (52,6)	56	49,6	
Sí	101 (47,0)	37	36,6	
Desconocido	1 (0,5)	0	0,0	
*Ventilación*				*0,410*
No	3 (1,4)	2	66,7	
Sí	212 (98,6)	91	42,9	
*Vacunación COVID-19*				*0,004*
No vacunado	16 (7,4)	6	37,5	
1 dosis	13 (6,0)	3	23,1	
2 dosis	61 (28,4)	19	31,1	
3 dosis	95 (44,2)	44	46,3	
4 dosis	30 (14,0)	21	70,0	
Total	215 (100)	93	43,3	

TAS: tasa de ataque secundaria.

La TAS global fue del 43,3%. En los contactos de 65 años o más la TAS fue superior respecto al resto grupos de edad (70 vs 35,2%; p=0,001), y también entre los que presentaban alguna enfermedad crónica (62,1 vs 36,3%; p=0,001). La TAS fue similar entre hombres y mujeres (40 vs 46,7%; p=0,324). En relación al uso de medidas preventivas no farmacológicas, el 35,8% afirmó haber utilizado mascarilla en el momento de la detección del caso de COVID-19 en el domicilio, el 87% tuvo una frecuencia de lavado de manos de más de tres veces al día y el 98,6% realizó una ventilación frecuente en el domicilio de al menos dos veces al día. Sin embargo, no se encontraron diferencias estadísticamente significativas para la TAS según el uso de las medidas preventivas no farmacológicas. El 86,6% de los contactos había recibido dos o más dosis de la vacuna contra la COVID-19; observando una mayor TAS (46,3% y 70%, respectivamente) para los contactos que habían recibido 3 y 4 dosis (Tabla 1).

### Nivel de conocimientos y actitudes

Los conocimientos y actitudes sobre la COVID-19 y sus medidas preventivas fueron evaluados en los 172 contactos mayores de edad.

**Figura 1:**
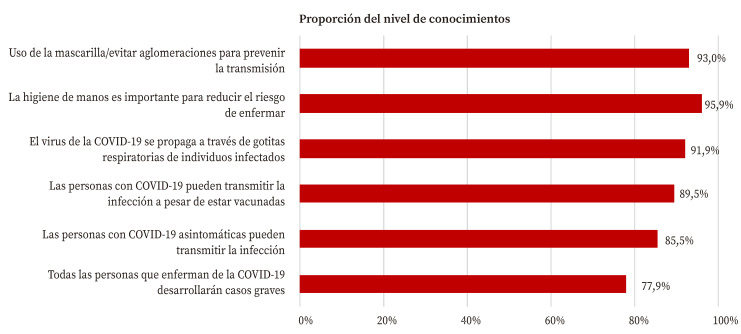
Proporción de conocimientos correctos sobre la COVID-19 de los contactos domiciliarios de casos de COVID-19.

En general, se observó un conocimiento correcto sobre la enfermedad y sus medidas preventivas (Fig. 1). Un 77,9% de los contactos indicaron correctamente que no todas las personas que enferman van a desarrollar casos graves, y un 85,5% indicó que las personas con COVID-19 asintomáticas pueden transmitir la infección. Además, el 89,5% señaló que se puede transmitir la COVID-19 a pesar de estar vacunado, el 91,9% conocía la forma de transmisión, el 95,9% tuvo un conocimiento correcto sobre la importancia de la higiene de manos y el 93% sobre el uso de la mascarilla.

**Figura 2:**
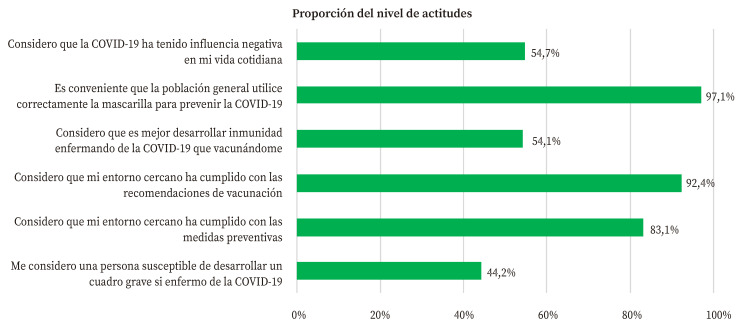
Proporción de actitudes positivas frente a la COVID-19 de los contactos domiciliarios de casos de COVID-19.

Al analizar las actitudes frente a la COVID-19 (Fig. 2) se observó que un 83,1% consideraba que su entorno cercano había cumplido con las medidas preventivas no farmacológicas y que un 92,4% había seguido las recomendaciones de vacunación. El 54,1% de los contactos domiciliarios consideró que era mejor desarrollar inmunidad enfermando de COVID-19 que vacunándose. El 44,2% se consideró una persona susceptible a desarrollar una forma grave. Solo el 54,7% de los contactos respondió que la COVID-19 tuvo una influencia negativa en su vida cotidiana.

## DISCUSIÓN

En este estudio se analizaron el nivel de conocimientos, actitudes y la utilización de las medidas preventivas no farmacológicas y de vacunación frente a la COVID-19 tras la fase aguda de la pandemia, en la que se produjo una relajación de dichas medidas preventivas en España. Nuestros resultados evidencian un adecuado uso de las medidas preventivas no farmacológicas, en los que más del 80% de los contactos domiciliarios realizó un lavado de manos adecuado y de ventilación frecuente del domicilio. Sin embargo, tan solo un 35,8% de los contactos domiciliarios declaró haber utilizado mascarilla tras la aparición del caso de COVID-19 en el domicilio.

Se observa un adecuado nivel de conocimientos y actitudes positivas frente a la COVID-19 en los contactos domiciliarios de un caso de COVID-19 confirmado. En el momento de la aparición del caso en el domicilio, más del 85% de los contactos señalaron correctamente la forma de transmisión de la COVID-19, y más del 93% tenían un conocimiento correcto de las medidas preventivas no farmacológicas de uso de mascarilla y lavado de manos. Estos resultados respaldan lo observado en el estudio COSMO-SPAIN, realizado en población española al inicio de la pandemia, en el que señalaba que entre el 80%-90% de los participantes indicaron correctamente las formas de contagio y el uso de la mascarilla para evitar la propagación de la COVID-19, aunque rondas posteriores muestran cómo el nivel de conocimiento sobre la COVID-19 fue descendiendo progresivamente a lo largo del año 2022^11^^,^^12^.

A pesar del alto nivel de conocimientos y actitudes positivas observadas en nuestro estudio, es importante considerar que, durante el periodo 2022-2023, muchas de las medidas preventivas no farmacológicas frente a la COVID-19, como la utilización de la mascarilla o el distanciamiento social, se fueron flexibilizando, lo cual podría verse reflejado en nuestros resultados. Dos meta-análisis recientes muestran resultados heterogéneos en relación a los niveles de conocimientos y actitudes^7^^,^^13^, señalando que estos niveles variaban entre un 35-95% y un 26-96%, respectivamente. En ambos estudios, los autores señalaron que la heterogeneidad de los resultados podría deberse a factores relacionados al nivel educativo, área de residencia, entre otros.

En el presente estudio, la mitad de los participantes indicó que es mejor desarrollar inmunidad enfermando de COVID-19 que vacunándose. Sin embargo, menos del 45% de los contactos domiciliarios presentaron una actitud correcta en relación a la susceptibilidad a desarrollar un caso grave, al igual que en la última ronda del estudio COSMO-SPAIN, en el que se observó que la percepción del riesgo había disminuido^12^. Cabe señalar que la percepción del riesgo de enfermar puede influir en los comportamientos relacionados con la salud y modificar las conductas de riesgo^14^. Por lo tanto, es necesario estudiar los conocimientos y actitudes de los grupos de alto riesgo, especialmente los que padecen enfermedades crónicas, para prevenir, controlar y mitigar los efectos de la COVID-19^15^.

Este estudio tiene algunas fortalezas y limitaciones. Se trata de un estudio descriptivo y el número de participantes es pequeño debido a la baja incidencia de casos de COVID-19 durante la fase de remisión de la pandemia, lo que no ha permitido obtener comparaciones entre el nivel de conocimientos y actitudes según características de los contactos. No se ha considerado necesario desagregar los resultados por sexo porque la TAS fue similar entre hombres y mujeres. Sin embargo, se ha realizado un censo exhaustivo de los contactos domiciliarios con una tasa de participación del 75% que incluye participantes de dos regiones españolas. El estudio se llevó a cabo en un periodo sujeto a una relajación de las medidas preventivas no farmacológicas, del que no tenemos apenas evidencia sobre los aspectos evaluados en otros lugares. Al tratarse de un estudio basado en encuestas, los datos obtenidos han sido autorreferidos, por lo que podría estar afectado por un posible sesgo de recuerdo^16^. Sin embargo, los datos relativos a la presencia de comorbilidades y el estado de vacunación frente a la COVID-19 fueron verificados con los registros médicos electrónicos y los registros de vacunación.

En conclusión, los contactos domiciliarios de casos de COVID-19 muestran un correcto conocimiento y actitud positiva frente a la COVID-19 y sus medidas preventivas durante la fase de remisión de la pandemia, durante la cual hubo una relajación de las medidas preventivas no farmacológicas. Estos resultados proporcionan información relevante a los responsables sanitarios para la toma de decisiones en caso de un aumento de casos de COVID-19 y para el desarrollo de intervenciones específicas y eficaces frente a la circulación del SARS-CoV-2 o de una nueva emergencia sanitaria por virus respiratorios.
